# Systems Genetics of Optic Nerve Axon Necrosis During Glaucoma

**DOI:** 10.3389/fgene.2020.00031

**Published:** 2020-02-27

**Authors:** Andrew B. Stiemke, Eric Sah, Raven N. Simpson, Lu Lu, Robert W. Williams, Monica M. Jablonski

**Affiliations:** ^1^ Department of Genetics, Genomics and Informatics, The University of Tennessee Health Science Center, Memphis, TN, United States; ^2^ Department of Ophthalmology, The Hamilton Eye Institute, The University of Tennessee Health Science Center, Memphis, TN, United States

**Keywords:** glaucoma, optic nerve damage, systems genetics, genetic reference panel, retinal ganglion cell, neurodegeneration, BXD recombinant inbred strains

## Abstract

In this study, we identify genomic regions that modulate the number of necrotic axons in optic nerves of a family of mice, some of which have severe glaucoma, and define a set of high priority positional candidate genes that modulate retinal ganglion cell (RGC) axonal degeneration. A large cohort of the BXD family were aged to greater than 13 months of age. Optic nerves from 74 strains and the DBA/2J (D2) parent were harvested, sectioned, and stained with p-phenylenediamine. Numbers of necrotic axons per optic nerve cross-section were counted from 1 to 10 replicates per genotype. Strain means and standard errors were uploaded into GeneNetwork 2 for mapping and systems genetics analyses (Trait 18614). The number of necrotic axons per nerve ranged from only a few hundred to more than 4,000. Using conventional interval mapping as well as linear mixed model mapping, we identified a single locus on chromosome 12 between 109 and 112.5 Mb with a likelihood ratio statistic (LRS) of ~18.5 (*p* genome-wide ~0.1). Axon necrosis is not linked to locations of major known glaucoma genes in this family, including *Gpnmb*, *Tyrp1, Cdh11, Pou6f2,* and *Cacna2d1*. This indicates that although these genes contribute to pigmentary dispersion or elevated IOP, none directly modulates axon necrosis. Of 156 positional candidates, eight genes—*CDC42 binding protein kinase beta* (*Cdc42bpb*); *eukaryotic translation initiation factor 5* (*Eif5*); *BCL2-associated athanogene 5* (*Bag5*); *apoptogenic 1, mitochondrial* (*Apopt1*); *kinesin light chain 1* (*Klc1*); *X-ray repair cross complementing 3* (*Xrcc3*); *protein phosphatase 1, regulatory subunit 13B* (*Ppp1r13b*); and *transmembrane protein 17*9 (*Tmem179*)—passed stringent criteria and are high priority candidates. Several candidates are linked to mitochondria and/or axons, strengthening their plausible role as modulators of ON necrosis. Additional studies are required to validate and/or eliminate plausible candidates. Surprisingly, IOP and ON necrosis are inversely correlated across the BXD family in mice >13 months of age and these two traits share few genes among their top ocular and retinal correlates. These data suggest that the two traits are independently modulated or that a more complex and multifaceted approach is required to reveal their association.

## Introduction

Glaucoma is a complex, multifactorial, neurodegenative disease that targets axons of retinal ganglion cells (RGCs) and is the leading cause of irreversible blindness worldwide (Standefer, 2004). The various subtypes of glaucoma—primary open angle (POAG), primary angle closure (PACG), and normal tension glaucoma—share the common clinical pathologies of RGC degeneration, RGC axonal damage, and subsequent loss of vision (Allingham et al., 2009). Intraocular pressure (IOP) is a major contributing factor to glaucoma, although elevated IOP is not detectable in all cases. In POAG, optic nerve (ON) axonal damage and IOP are highly heritable, and the genetic risk of elevated IOP and POAG are partially shared (Van Koolwijk et al., 2007; Carbonaro et al., 2008). However, many of the gene variants and loci associated with POAG are not linked functionally with the control of IOP (Thorleifsson et al., 2010). It is therefore highly likely that a subset of gene variants modulate the risk of RGC axon damage in an independent manner, and thereby directly affect the onset of glaucoma, rate of axon necrosis, and response to therapeutic interventions such as nicotinamide (Williams et al., 2017). While multiple genes, proteins, and transcription factors have been identified that are associated with axon death [e.g. *BAX* (BCL2-associated X protein), *TRX* (thioredoxin), *HIF-1α* (hypoxia-inducible factor-1α), *SNCG* (γ-synuclein), *ATF4* (activating transcription factor 4), *DDIT3* (DNA damage inducible transcript 3), and *BCAT1* (branched chain amino acid transaminase 1) (Libby et al., 2005a; Soto et al., 2008; Munemasa and Kitaoka, 2013; Yasuda et al., 2014; Cheng et al., 2017), the causative genes and variants remain unknown for the majority of glaucoma cases. Identification of additional sequence variants, and associated molecular and cellular processes that modulate glaucoma endophenotypes in animals and humans can definitely provide critical insights, as well as new targets for therapeutic intervention (Chintalapudi et al., 2017).

Systems genetics is a scientific approach that collectively analyzes large cohorts of isogenic and fully sequenced individuals to evaluate relationships among SNPs, other sequence variants, molecular endophenotypes (Geisert et al., 2009; Williams and Williams, 2017), cellular function and properties (e.g. RGC types and numbers (Williams et al., 1998)), rates of axonal degeneration, and classical clinical phenotypes such as IOP (Chintalapudi et al., 2017; King et al., 2018a), visual field loss, age, sex, and environmental cofactors. In the present study, we apply this general approach to ongoing studies of glaucoma using the BXD family of mice. The BXDs are a very large family (n = 152) of fully sequenced and isogenic recombinant inbred (RI) strains derived by crossing C57BL/6J (B6, the mouse genome reference strain) and DBA/2J (D2, a glaucoma-prone strain). All progeny of this cross have now been sibmated for more than 20 generations. The BXDs are an unrivaled resource for ocular systems genetics primarily because phenome data, including remarkably deep data on the primary visual system from the cornea through to the cortex, has been assembled for many of the BXDs [e.g., cornea and lens (Zhou and Williams, 1999; King et al., 2018b); RGCs (Williams et al., 1998); lateral geniculate nucleus (Seecharan et al., 2003); and visual cortex (Heimel et al., 2008)]. All have been fully genotyped (Peirce et al., 2004). Five to six million variants segregate among the BXD family (Wang et al., 2016), giving them a genetic complexity approaching that of human populations, but with the major advantage of being able to replicate any and all individuals and thereby explore gene-by-environment, gene-by-age, and gene-by-therapy effects (Chintalapudi et al., 2017). Genome and phenome data sets for this family have been assembled in what is essentially an open source electronic health care database (gn2.genenetwork.org) that is now widely used as an experimental platform for personalized and probabilistic medicine (Koutnikova et al., 2009; Jablonski et al., 2011; Lu et al., 2011a; Lu et al., 2011b; Swaminathan et al., 2013; Chintalapudi et al., 2015; Lu et al., 2016; Chintalapudi et al., 2017).

In the current study, we have systematically quantified the severity of ON damage using a large subset of the BXD family across five age cohorts. Using stringent, stepwise refinement based on expression quantitative trait locus (eQTL) mapping, correlation analyses (direct Pearson test), and analysis of polymorphisms, we mapped an axon necrosis modifier locus and have narrowed the list of genes to eight high priority candidates. Lastly, because both ON damage and elevated IOP are endophenotypes associated with glaucoma, we tested whether or not the modulation of axon necrosis and IOP are shared. Contrary to our expectations, they are not. We show that these two traits are independently controlled and that molecular networks share minimal overlap, further demonstrating the genetic complexity of glaucoma and its associated endophenotypes.

## Materials and Methods

### Animals

We obtained ONs from 74 BXD strains, the DBA/2J parental strain (*n* = 347) between 13 and 40 months of age. All procedures involving mice were approved by the Animal Care and Use review board of UTHSC and followed the Association of Research in Vision and Ophthalmology (ARVO) Statement for the Use of Animals in Ophthalmic and Vision Research, in addition to the guidelines for laboratory animal experiments (Institute of Laboratory Animal Resources, Public Health Service Policy on Humane Care and Use of Laboratory Animals). Mice were housed under cyclic light (12 h on:12 h off) with 35% humidity in a specific-pathogen free (SPF) facility at UTHSC and were allowed free access to water and food. Of the 347 animals 104 were males (average age of 18.2 months) and 243 were females. (average age of 18.2 months).

### Quantification of the Number of Necrotic Axons in the Optic Nerve and Heritability Calculation

**Figure 1:**
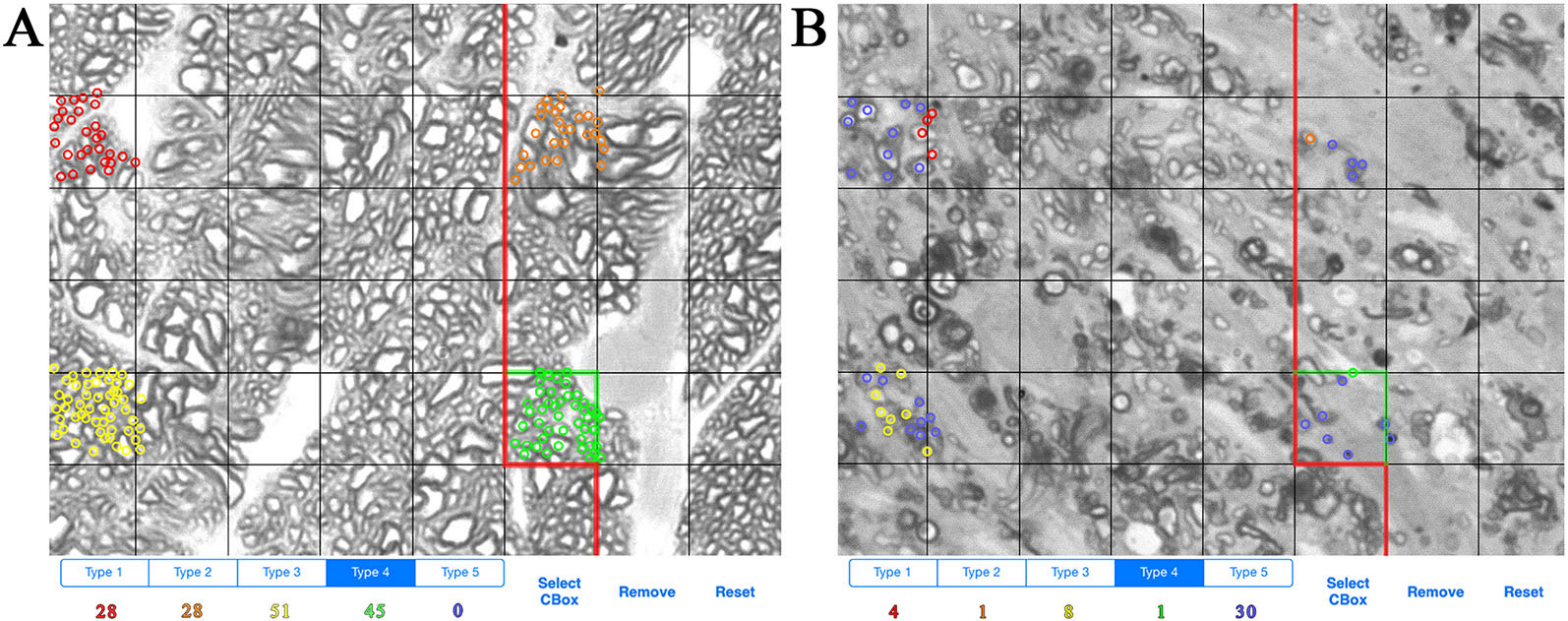
Example of axon counting and categorization method. **(A)** Example of axon counts from the optic nerve of a BXD36 mouse aged >13 months. Each color (red, orange, yellow, green) is used to count individual live axons in a single box unit. **(B)** Example of axons counts from the optic nerve of a BXD29 mouse aged >13 months. Red, orange, yellow, and green indicate live axons, while blue indicates necrotic axons.

ONs were harvested immediately after sacrifice and placed in Smith-Rudt fixative for 24 h. After postfixation with 1% osmium tetroxide, nerves were dehydrated and embedded in Epon 812 using our published methods (Jablonski et al., 2005). One-micron thick sections were cut from the proximal portion of ONs and were stained with 1% p-phenylenediamine (PPD) to stain the myelin sheath of all axons, and the axoplasm of necrotic axons. A single cross section of the ON was selected for analysis. ONs were photographed at a magnification of 1,200x using a 100x oil-immersion objective. ON axons were designated as live or necrotic based on the relative PPD-staining of the axoplasm and counted with ImagePad software (Templeton et al., 2014) using a minimum of 12 fields (7.49 micron x 7.49 micron) evenly spaced across the ON to calculate the number and density of healthy and degenerating axons (**Figures 1A, B**). Data for all ONs were compiled and exported to GeneNetwork (www.genenetwork.com). Data are presented as mean ± SEM. The average number of necrotic axons per ON and density of necrotic axons are available on GeneNetwork as phenotype record IDs 18614 and 21437, respectively.

To determine the degree to which variation in the number of necrotic axons was due to genetic effects rather than technical or environmental factors, we calculated heritability (*h* RIx̅) using the formula of Belknap (1998).

### Identification of Gene Candidates That Modulate the Number of Necrotic Axons Per ON

**Figure 2:**
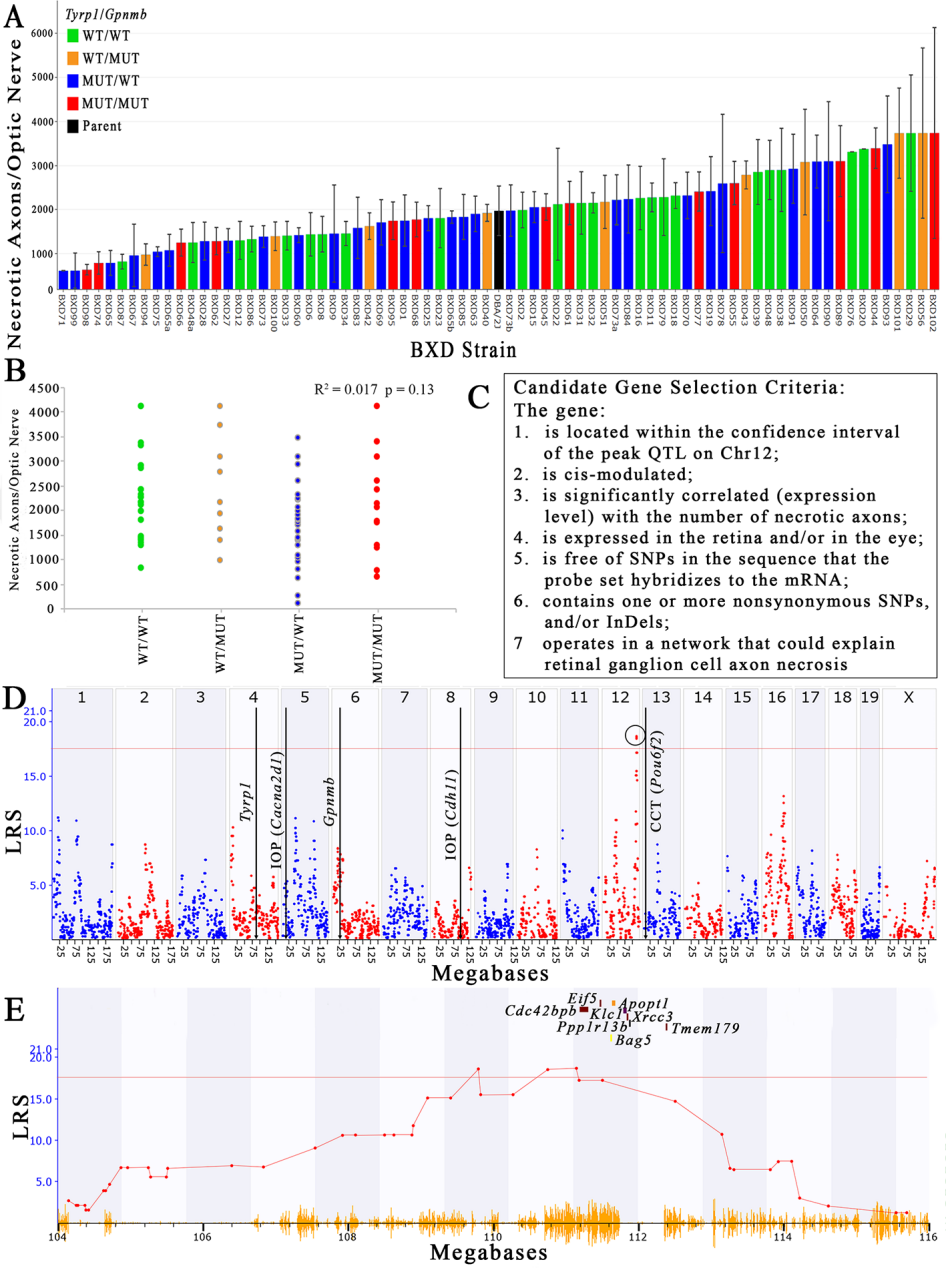
Identification of a QTL that modulates the number of necrotic axons in the optic nerve. **(A)** The number of necrotic axons varies among the BXD strains. Necrotic axons of BXD strains carrying wild type alleles of *Tyrp1* and *Gpnmb* (WT/WT; green bars), wild type *Tyrp1* and mutant *Gpnmb* (WT/MUT; orange bars), mutant *Tyrp1* and wild type *Gpnmb* (MUT/WT; blue bars), and mutant alleles of both genes (MUT/MUT; red bars) are shown. n = 75 strains, age = 13-30 months. Values denote mean number of necrotic axons per nerve. **(B)** *Tyrp1* and *Gpnmb* haplotypes do not influence axon necrosis in BXD mice. The scatter plot shows average number of necrotic axons per nerve of BXD strains carrying wild type alleles of *Tyrp1* and *Gpnmb* (WT/WT; green dots), wild type allele of *Tyrp1* and mutant allele of *Gpnmb* (WT/MUT; orange dots), mutant allele of *Tyrp1* and wild type allele of *Gpnmb* (MUT/WT; blue dots), and mutant alleles of both genes (MUT/MUT; red dots) (n = 75, p = 0.13, age = 13-30 months). P-value was calculated using an ANOVA. **(C)** Stringent selection criteria for selecting candidate genes. **(D)** A significant QTL for the number of necrotic axons per optic nerve is present on chromosome 12 between 109 and112.5Mb. This is distinct from the locations of *Tyrp1* and *Gpnmb* in the genome. It is also distinct from loci that have been demonstrated to modulate IOP (Chintalapudi et al., 2017; King et al., 2018a) and central corneal thickness (King et al., 2018b). **(E)** Expanded view of the Chr 12 QTL showing the location of the eight strongest positional candidate genes.

Candidate genes that modulate the number of necrotic axons in the optic nerve were identified using our stringent inclusion criteria and the tools available in GeneNetwork. To rank candidate genes in the significant locus, we used the following criteria adapted from Chintalapudi et al. (2017), **Figure 2C**:

1. the gene is located within the confidence interval of the peak QTL obtained from simple interval mapping;
2. the gene is cis-modulated in the retina and/or the eye;
3. the expression level of the gene in the retina and/or the eye across BXD strains is significantly correlated with the number of necrotic axons;
4. the gene has an expression level in the retina and/or the eye of 6.8 or greater;
5. the mRNA sequence to which the probe sets hybridize is free of single nucleotide polymorphisms (SNPs);
6. the gene harbors at least one nonsynonymous SNP, transcript variant and/or insertion/deletion (InDel); and
7. the gene operates in a network that can explain a plausible role in the health or damage to the optic nerve.

### QTL Mapping

The number of necrotic axons in the ON and the density of necrotic axons in the ON in BXD mice greater than 13 months of age are publically available as BXD published phenothype record IDs 18614 (http://www.gn2.genenetwork.org/show_trait?trait_id=18614&dataset=BXDPublish) and 21437 (http://www.gn2.genenetwork.org/show_trait?trait_id=21437&dataset=BXDPublish), respectively. To identify chromosomal regions that modulate these traits, we used the GEMMA mapping function in GeneNetwork 2 using default settings (MAF > 0.05 and using LOCO) and the more conventional Haley-Knott interval mapping implemented in GeneNetwork 1. The former method corrects for kinship relations among BXD strains (e.g., BXD73, BXD73a, BXD73b), whereas the Haley-Knott method does not factor in kinship. However, the latter method is computationally much faster and compatible with both permutation and bootstrap resampling. Before mapping, we reviewed data carefully and determined that that the distribution of trait values was close to normal. One strain (BXD71) had an unusually low number of necrotic axons per ON (< 300 per nerve) and three strains (BXD102, BXD56, and BXD29) had unusually high numbers of necrotic axons per ON (from 4,000 to 6,000). Values for these four strains were winsorized as recommended by Tukey (1962) prior to mapping as follows: the value for BXD102 was winsorized from 5,825 to 3,736; BXD56 was winsorized from 5,552 to 3,735; BXD29 was winsorized from 4,121 to 3,734; and BXD71 was winsorized from 376 to 616.

The major locus on Chr 12, that is the focus of this study, is robust with respect to mapping algorithm (i.e., GEMMA or Haley-Knott), genotype files, and is the only locus that achieves genome-wide significance. We tested the robustness of this locus using our data files in regard to the following scenarios: raw data without winsorizing; data after winsorizing as described above;and censoring the five outlier strains.

### Cis-Modulation and Probe Set Criteria

Gene expression was used as a microtrait to map regulatory eQTLs for the differences in mRNA expression levels in the family of BXD lines. Retina and whole eye transcript data from BXD strains available on GeneNetwork as Full HEI Retina V6.2 (Apr10) and Eye M430v2 (Sep08) RMA, respectively (Freeman et al., 2011) were used to identify cis-regulated genes within our interval of interest using our published methods (Chintalapudi et al., 2017). A gene was considered cis-modulated if its associated marker was localized within a 10 Mb window of its position in the genome. We also included among our candidates, genes that had sequence variants that may alter the function of the gene product, yet lack cis-modulation. The same transcript datasets were used to identify genes whose expression correlated with the number of necrotic axons. Only probe sets that did not bind to genomic regions containing SNPs were used to avoid hybridization artifacts that may result in artificial differences in expression due to technical error, rather than biological variance. All probes were verified by the BLAST-like alignment tool (BLAT) [UCSC Genome Browser (Casper et al., 2018)].

### Correlation and Network Analyses

Correlation values for initial candidate gene selection were determined using Pearson correlation coefficients of the trait. To be considered further, a gene was required to be among the top 1,000 correlates of the number of necrotic axons in the ON. The expression level of each cis-modulated candidate was determined using the same Full HEI Retina V6.2 (Apr10) or Eye M430v2 (Sep08) RMA databases (Freeman et al., 2011). Genes with expression levels greater than 6.8 were maintained among the list of candidates. Genes with expression levels less than 6.8 were considered below the level of detection, and therefore not expressed in the tissue. Each cis-regulated candidate was evaluated to identify those candidates that harbored nonsynonymous SNPs between B6 and D2 parents. The full array of SNPS, InDels and transcript variants were determined by querying the University of California Santa Cruz Genome Browser (http://ucscbrowserbeta.genenetwork.org/cgi-bin/hgTracks?org=mouse).

To determine the network within which each positional candidate gene functions, we identified the top 1,000 correlates of each candidate. Those correlates with expression levels >6.8 were used to perform gene ontology (GO) analysis using the cellular component database of WebGesalt (Wang et al., 2017). The categories that reached statistical significance and were terminal branches of the tree were plotted as bar graphs.

Because both IOP and ON damage are endophenotypes associated with glaucoma, we plotted these traits from the >13 month cohort as a scatterplot and determined their correlation. To determine if these traits share correlates and molecular networks, a list of the top 2,000 gene correlates of each trait were generated using both retina and whole eye databases. Both lists were filtered to include only those with expression levels >6.8. The number and identity of shared genes were determined.

## Results

### Optic Nerve Necrosis Across the BXD Family

The number of necrotic axons per nerve varies 15-fold between the extremes in the older (> 13-month) BXD strains, and ranges from a low of 276 dead axons/nerve in BXD71 to a high of 4,122 ± 2,392 dead axons/nerve in BXD102 (**Figure 2A**), which equates to a percentage range of 0.3% to 12.7% (data not shown). The conventional h^2^ calculation of heritability of the number of necrotic axons per ON is 36%, while the h^2^_Rix̄_ calculation of heritability (Belknap, 1998) is 58%, both of which are in the range of other ocular traits for which we have successfully used QTL mapping (Jablonski et al., 2011; Lu et al., 2011b; Swaminathan et al., 2013; Lu et al., 2016). The haplotypes of *Tyrp1* and *Gpnmb* for each strain are distributed across the plot when presented as rank ordered means. When separated into individual haplotypes at *Tyrp1* and *Gpnmb*, there is no statistical difference in the number of necrotic axons (R^2^ = 0.017 and p = 0.13; **Figure 2B**).

We do not have sufficient replication within all BXD strains to consider strain-specific sex differences, however across the BXD family, the average number of necrotic axons in ONs obtained from males and females is 1,989 ± 1,329 and 2,053 ± 1,556, respectively (p = 0.72). For a subset of 23 BXD strains, we have data for two or more mice of both sexes, which allowed us to evaluate the effect of sex within a strain. A two-sample t-test comparing data obtained from males and females of these 23 strains yielded a p-value of 0.36.

The incidence of axon necrosis is expected to be a positive function of age. To explore this theory, we performed a regression analysis of the number of necrotic axons per ON versus age, fit using either age in months or the logarithm of age. We determined that for each one month increment above 13 months of age, the number of necrotic axons increases by 34 (R^2^ = 0.0066; **Supplemental Figure 1**). This factor accounts for only 0.66% of the total variance among all 347 optic nerves.

Using both the simple interval mapping function of GeneNetwork 1 and the GEMMA linear mixed model function of GeneNetwork 2, the variability in the number of necrotic axons in the ON maps to Chr 12 between 109 Mb and 112.5 Mb. Moreover, the location of the peak linkage is not sensitive to inclusion or exclusion of values or to winsorizing. While the peak linkage score varies somewhat—from a low of about 15.5 using Haley-Knott methods to 18.5 using GEMMA (**Figure 2D**), it is the only peak with genome-wide significance under all mapping strategies. The Chr 12 locus has an additive effect of about +380 necrotic axons per *D* allele and nominally accounts for about 20% of the variance among strain means. Using the GEMMA algorithm, a single peak on Chr 16 reached a suggestive threshold, yet it failed to reach genome-wide significance. No other peak reached the suggestive threshold of ~11 (**Figure 2D**).

An identical Chr 12 locus is present when mapping the density of necrotic axons in the ON using the Haley-Knott algorithm (**Supplemental Figure 2**), strengthening the robustness of this finding.

Using data derived from younger aged mice (not shown), a similar quantitative trait locus (QTL) peak is not present, suggesting that the gene variant(s) responsible for modulating axon necrosis contribute to ON degeneration primarily at older ages, which is consistent with the age of onset of noncongenital glaucomas. Additionally, the peak locus does not overlap either the *Tyrp1* or *Gpnmb* genes, both of which harbor well documented mutations in the *D* haplotype that contribute to pigmentary dispersion glaucoma (Anderson et al., 2002). These findings demonstrate ON axon necrosis is not linked to either *Tyrp1* or *Gpnmb* and is therefore independent of two of the main genetic mutations known to contribute to pigmentary dispersion glaucoma in the D2 mouse. Interestingly, the chromosome 12 locus also does not overlap with other loci recently identified in the BXD family that modulate IOP (Chintalapudi et al., 2017; King et al., 2018a) or central corneal thickness (CCT, (King et al., 2018b)), both of which are risk factors for glaucoma (Investigators, 2000; Gordon et al., 2002; Medeiros et al., 2003; Group, 2007).

### Selection of Positional Candidates in the Chromosome 12 Locus

There are 156 position candidate genes within the QTL on chromosome 12 between 109 Mb and 112.5 Mb (**Supplemental Table 1**). Of those, 11 genes are cis-modulated in the retina and nine genes are cis-modulated in the eye. Four genes are in cis-modulated in both tissues (**Supplemental Table 2**). After applying our remaining selection criteria, only eight candidates remain as plausible modulators of axon necrosis, specifically: *CDC42 binding protein kinase beta* (*Cdc42bpb*); *eukaryotic translation initiation factor 5* (*Eif5*); *BCL2-associated athanogene 5* (*Bag5*); *apoptogenic 1, mitochondrial* (*Apopt1*); *kinesin light chain 1* (*Klc1*); *X-ray repair cross complementing 3* (*Xrcc3*); *protein phosphatase 1, regulatory subunit 13B* (*Ppp1r13b*); and *transmembrane protein 17*9 (*Tmem179*) (**Figure 2E**). No other candidates fulfil all of our inclusion criteria. **Table 1** provides a list of all cis-modulated positional candidate genes in the retina and/or eye, and indicates which selection criteria each gene fulfils. One gene—*WD repeat domain 25* (*Wrd25*)—has fulfilled all but one inclusion criteria and therefore is not considered as a top positional candidate. While *Wrd25* operates in a network associated with ON health, it is not among the top 1,000 correlates using either the retina or eye database and has been moved to a second tier candidate. After relaxing our inclusion criteria to also include genes that lacked cis-modulation, no additional candidates were identified (data not shown).

**Table 1:**
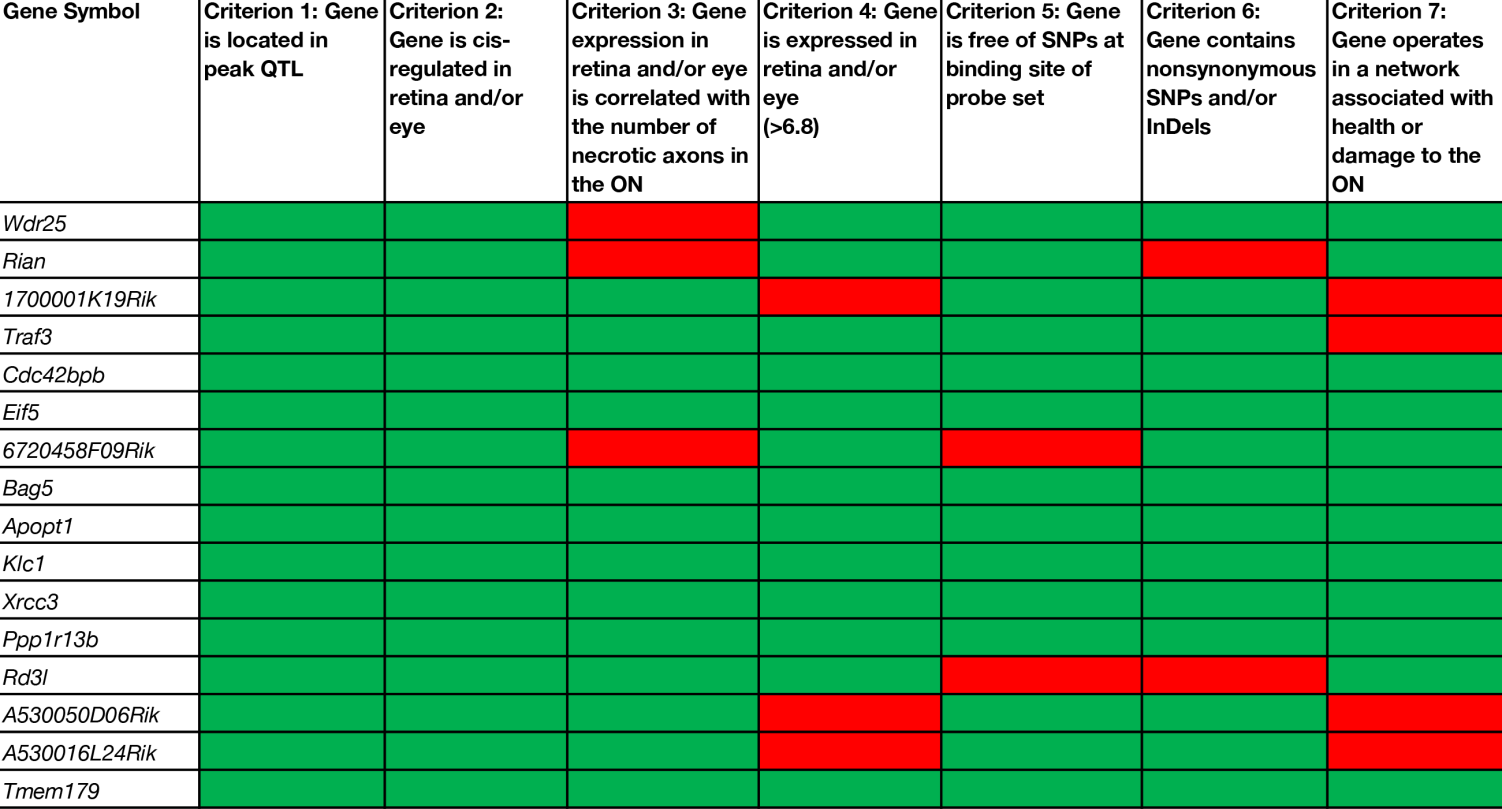
Selection criteria applied to cis-regulated positional candidates in the retina and/or eye.

### Evaluation of the Eight Positional Candidates That Fulfill All of Our Selection Criteria

**Figure 3:**
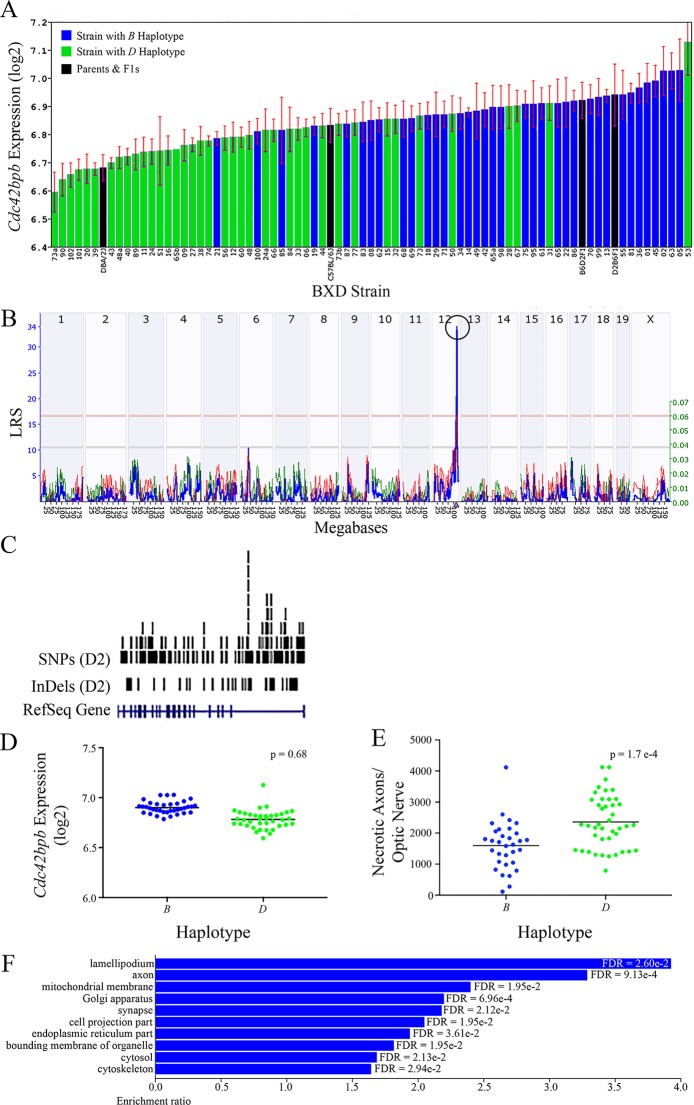
*Cdc42bpb* as a candidate gene for modulation of ON damage. **(A)** *Cdc42bpb* expression in the retina varies across BXD strains. The bars depict a range of expression values from 6.59 ± 0.07 and 7.13 ± 0.12. On the Y-axis, *Cdc42bpb* expression is presented on a Log2 scale. Parental strains and F1s are indicated by black bars. Mice harboring the B and D haplotypes of Cdc42bpb are indicated by blue and green bars, repectively. **(B)** Genetic mapping revealed a single highly significant *cis*-eQTL for *Cdc42bpb* on Chr 12. The purple triangle indicates the location of *Cdc42bpb* within the mouse genome. **(C)** UCSC Genome Browser illustration of gene structure, and location of SNPs and InDels in *Cdc42bpb* on Chr 12 of the mouse genome. Reference Sequence mRNA is represented in blue. **(D)** In this scatter plot, the haplotype of *Cdc42bpb* does not significantly influence the expression level of the gene in BXDs aged >13 months (p = 0.68). **(E)** In contrast, BXD strains that carry the *D* haplotype of *Cdc42bpb* have a significantly greater number of necrotic axons than those with the *B* haplotype (p = 1.7e-4). **(F)** Terminal statistically significant categories taken from GO tree analyses of the correlates of *Cdc42bpb* in the retina. False discovery rates are shown next to each bar. The X-axis denotes the ratio of observed genes by the number of expected genes in the GO network.

**Figure 4:**
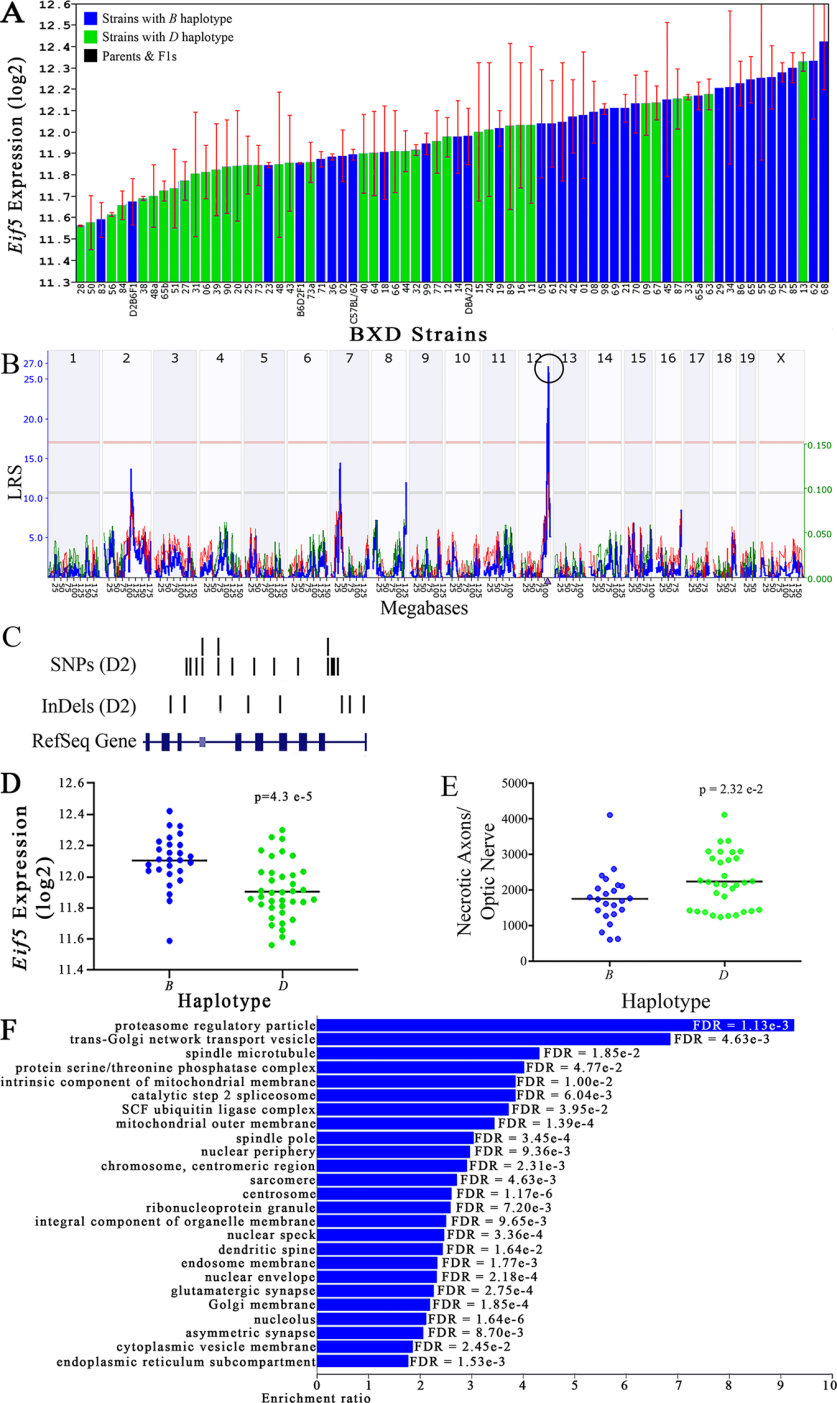
*Eif5* as a candidate gene for modulation of optic nerve damage. **(A)** *Eif5* expression in the eye, expressed on a Log_2_ scale, varies across BXD strains with a low of 11.56 ± 0.01 and a maximum of 12.42 ± 0.23. Parental strains and F1s are indicated by black bars. Mice with the *B* and *D* haplotypes are indicated by blue and green bars, respectively. **(B)** *Eif5* maps as a highly significant *cis*-eQTL on Chr 12. The purple triangle indicates the location of *Eif5* within the mouse genome. **(C)** UCSC Genome Browser illustration of gene structure, and location of SNPs and InDels in *Eif5*. Reference Sequence mRNA is represented in blue. **(D)** Mice with the *D* haplotype of *Eif5* have significantly lower expression levels of the gene than those with the B haplotype (p = 4.3e-5). **(E)** In contrast, strains that carry the *D* haplotype of *Eif5* have a significantly greater number of necrotic axons than those with the *B* haplotype (p = 2.32e-2). **(F)** Terminal statistically significant categories taken from GO tree analyses of the correlates of *Eif5* in the eye. False discovery rates are shown next to each bar. The X-axis denotes the ratio of observed genes by the number of expected genes in the GO network.

**Figure 5:**
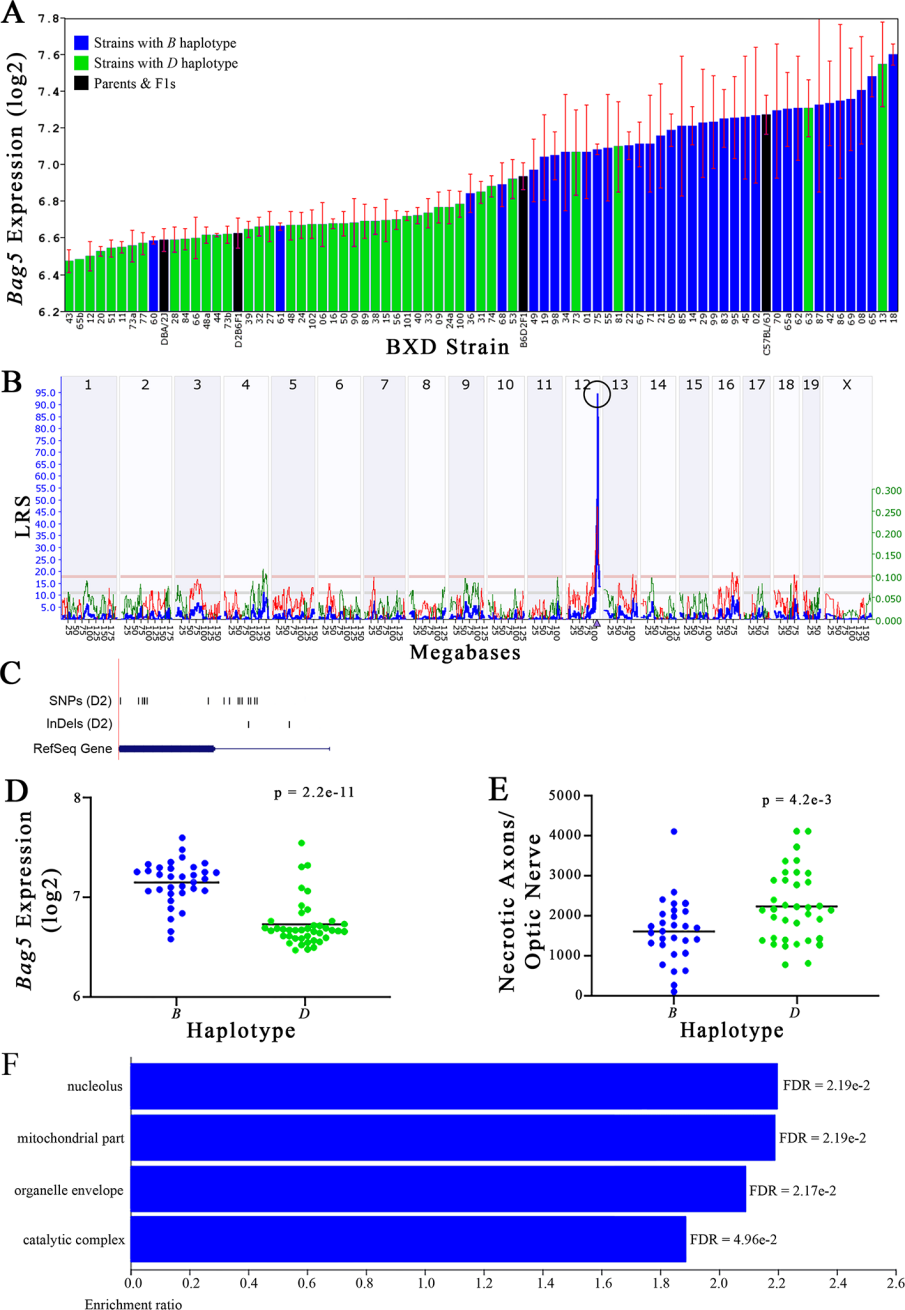
*Bag5* as a candidate gene for modulation of optic nerve damage. **(A)** *Bag5* expression in the retina, expressed on a Log_2_ scale, varies across BXD strains with a low of 6.47 ± 0.06 and a maximum of 7.60 ± 0.06. Parental strains and F1s are indicated by black bars. Mice with the *B* and *D* haplotypes are indicated by blue and green bars, respectively. **(B)** *Bag5* maps as a highly significant *cis*-eQTL on Chr 12. The purple triangle indicates the location of *Bag5* within the mouse genome. **(C)** UCSC Genome Browser illustration of gene structure, and location of SNPs and InDels in *Bag5*. Reference Sequence mRNA is represented in blue. **(D)** Mice with the *D* haplotype of *Bag5* have significantly lower expression levels of the gene than those with the *B* haplotype (p = 2.2e-11). **(E)** In contrast, strains that carry the *D* haplotype of *Bag5* have a significantly greater number of necrotic axons than those with the *B* haplotype (p = 4.2e-3). **(F)** Terminal statistically significant categories taken from GO tree analyses of the correlates of *Bag5* in the retina. False discovery rates are shown next to each bar. The X-axis denotes the ratio of observed genes by the number of expected genes in the GO network.

**Figure 6:**
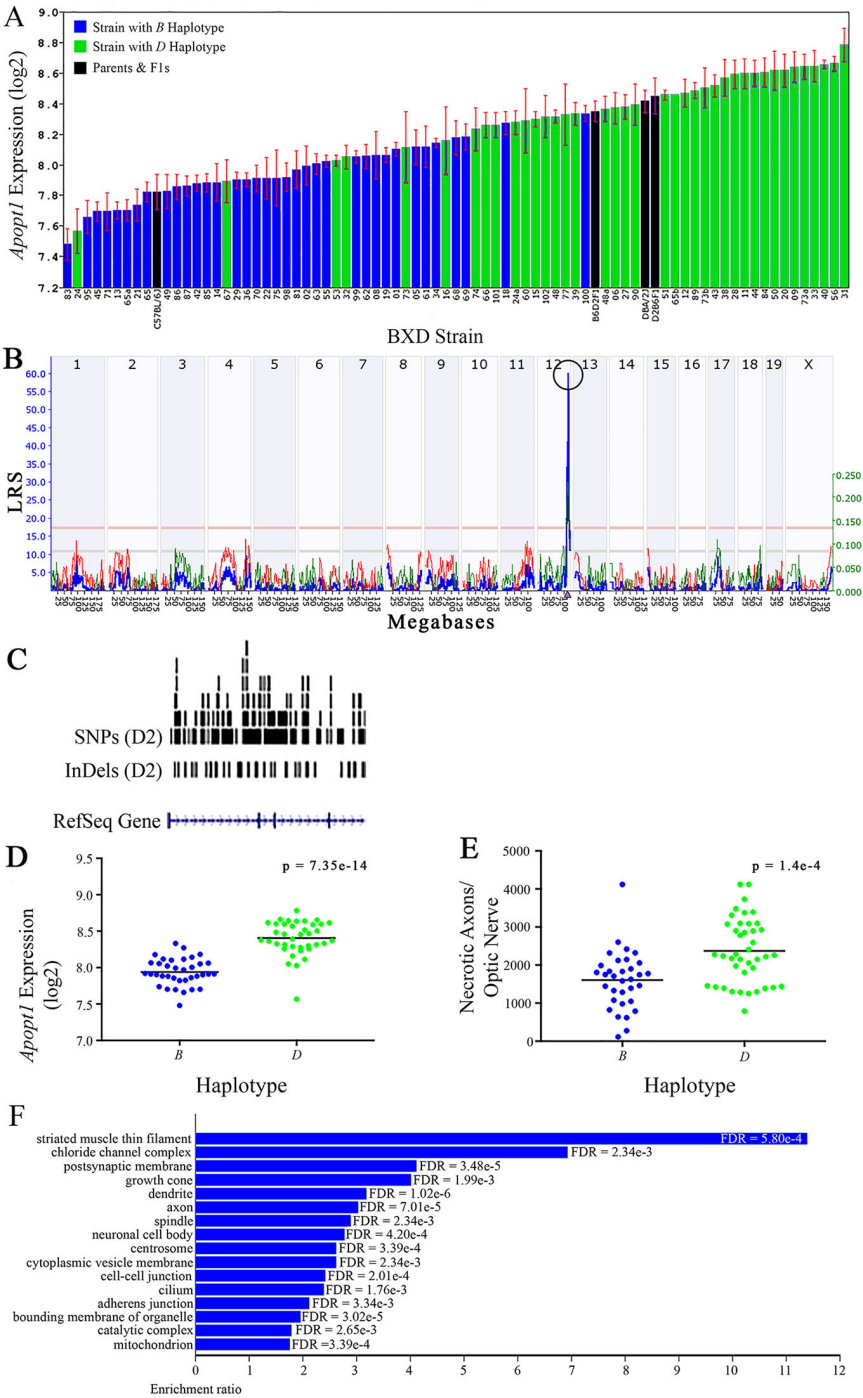
*Apopt1* as a candidate gene for modulation of optic nerve damage **(A)** *Apopt1* in the retina, expressed on a Log_2_ scale,varies across BXD strains from a minimum of 7.48 ± 0.10 and a maximum of 8.78 ± 0.11. Parental strains and F1s are indicated by black bars. Mice harboring the *B* and *D* haplotypes of *Apopt1* are indicated by blue and green bars, respectively. **(B)** Genetic mapping revealed a single highly significant cis-eQTL for *Apopt1* on Chr 12. The purple triangle indicates the location of *Apopt1* within the mouse genome. **(C)** UCSC Genome Browser illustration of gene structure, and location of SNPs and InDels in *Apopt1* of the mouse genome. Reference Sequence mRNA is represented in blue. **(D)** The *D* haplotype of *Apopt1* is significantly correlated with a higher level of gene expression in the retina (p = 7.35e-14). **(E)** Likewise, BXD strains that carry the *D* haplotype of *Apopt1* have a significantly greater number of necrotic axons than those with the *B* haplotype (p = 1.4e-4). **(F)** Terminal statistically significant categories taken from GO tree analyses of the correlates of *Apopt1* in the retina with false discovery rates shown next to each bar. The X-axis denotes the ratio of observed genes by the number of expected genes in the GO network.

**Figure 7:**
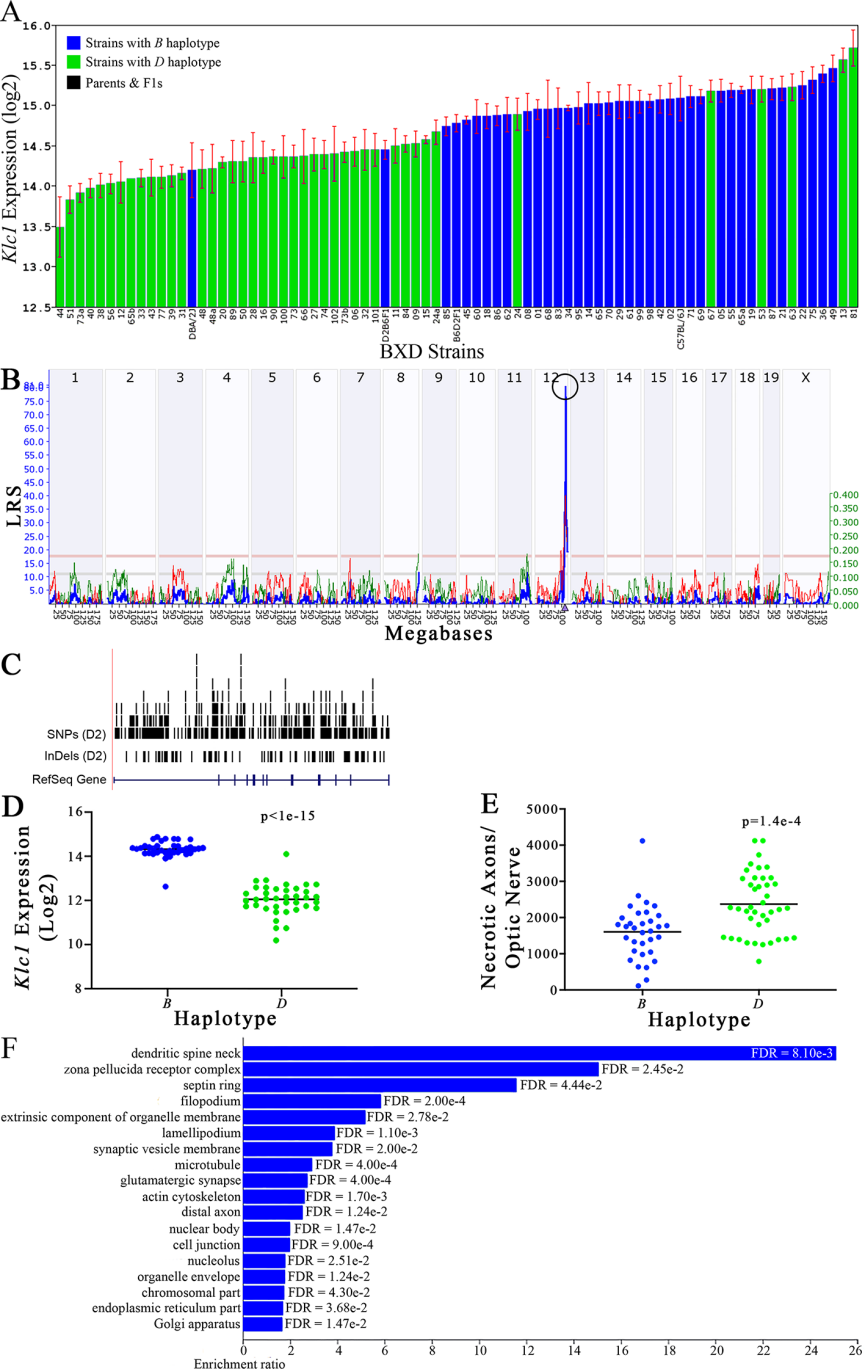
*Klc1* as a candidate gene for modulation of optic nerve damage. **(A)** *Klc1* expression in the eye, expressed on a Log_2_ scale, varies across BXD strains with a low of 13.50 ± 0.35 and a maximum of 15.73 ± 0.24. Parental strains and F1s are indicated by black bars. Mice with the *B* and *D* haplotypes are indicated by blue and green bars, respectively. **(B)** *Eif5* maps as a highly significant *cis*-eQTL on Chr 12. The purple triangle indicates the location of *Eif5* within the mouse genome. **(C)** UCSC Genome Browser illustration of gene structure, and location of SNPs and InDels in *Eif5*. Reference Sequence mRNA is represented in blue. **(D)** Mice with the *D* haplotype of *Eif5* have significantly lower expression levels of the gene than those with the *B* haplotype (p < 1e-15). **(E)** In contrast, strains that carry the *D* haplotype of *Eif5* have a significantly greater number of necrotic axons than those with the *B* haplotype (p = 1.4e-4). **(F)** Terminal statistically significant categories taken from GO tree analyses of the correlates of *Eif5* in the eye. False discovery rates are shown next to each bar. The X-axis denotes the ratio of observed genes by the number of expected genes in the GO network.

**Figure 8:**
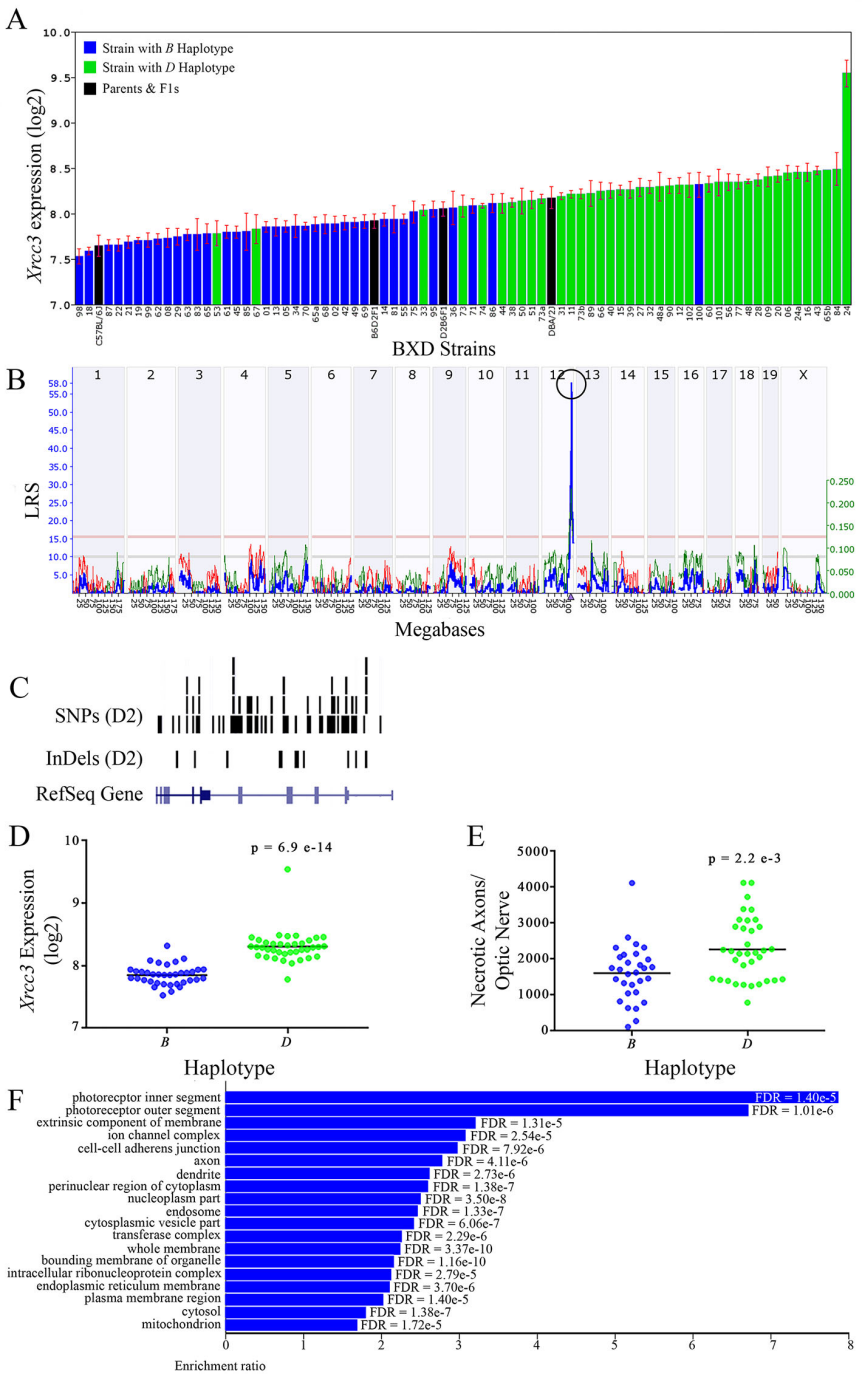
*Xrcc3* as a candidate gene for modulation of optic nerve damage. **(A)** *Xrcc3* expression in the retina varies across BXD strains. The bars depict a range of expression values from 7.53 ± 0.84 and a maximum of 9.54 ± 0.15. Parental strains and F1s are indicated by black bars. Mice harboring the *B* and *D* haplotypes of *Xrcc3* are indicated by blue and green bars, respectively. **(B)** A single highly significant *cis*-eQTL for *Xrcc3* is present on Chr 12. The purple triangle indicates the location of *Xrcc3* within the mouse genome. **(C)** Gene structure, and location of SNPs and InDels in *Xrcc3* of the mouse genome per UCSC Genome Browser. Reference Sequence mRNA is represented in blue. **(D)** The *D* haplotype of *Xrcc3* is significantly correlated with an elevated level of gene expression in the retina (p = 6.9e-14). **(E)** Similarly, BXD strains that carry the *D* haplotype of *Xrcc3* have a significantly greater number of necrotic axons than those with the *B* haplotype (p = 2.2e-3). **(F)** Terminal statistically significant categories taken from GO tree analyses of the correlates of *Xrcc3* in the retina with false discovery rates shown next to each bar. The X-axis denotes the ratio of observed genes by the number of expected genes in the GO network.

**Figure 9:**
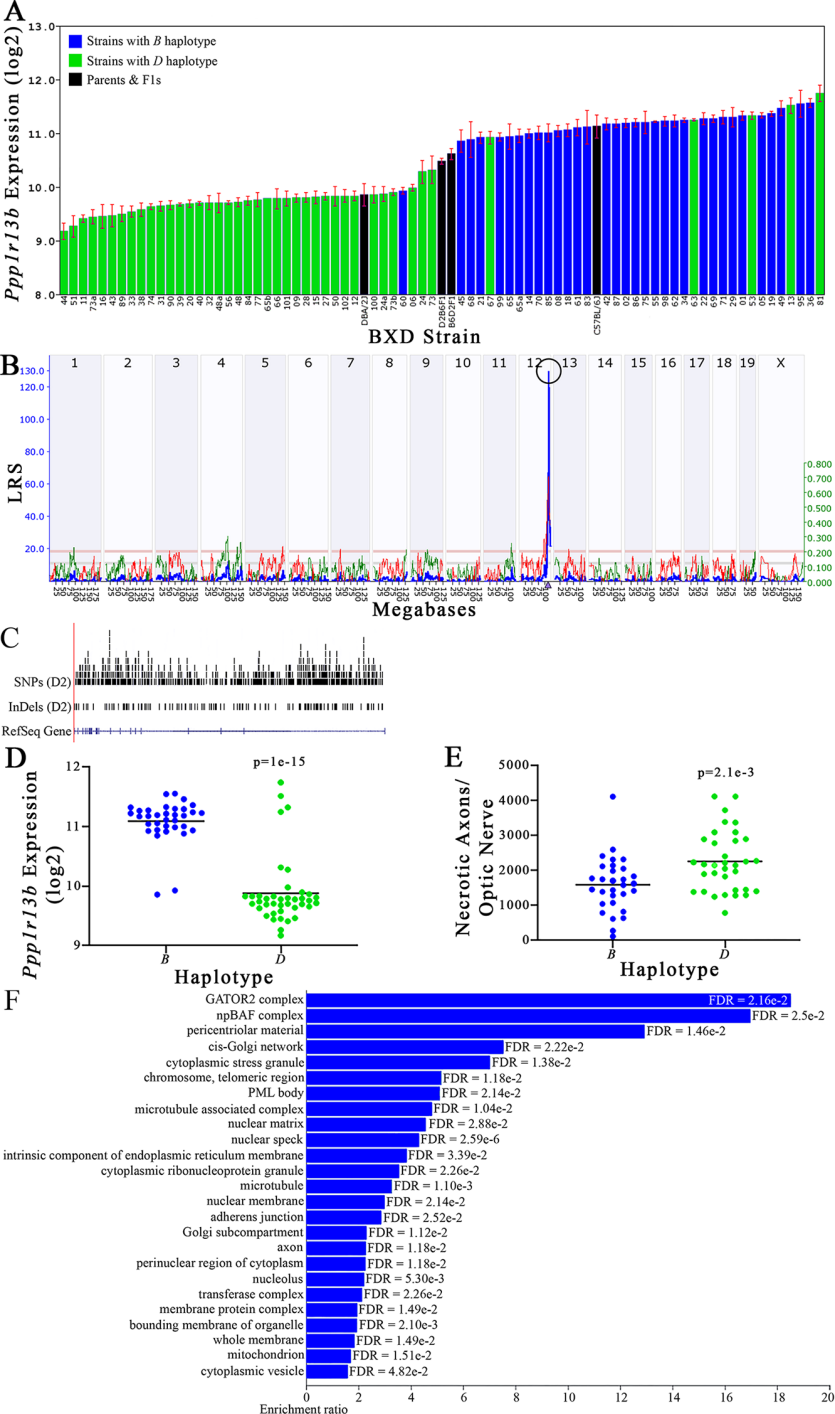
*Ppp1r13b* as a candidate gene for modulation of optic nerve damage. **(A)** *Ppp1r13b* expression in the retina, expressed on a Log_2_ scale, varies across BXD strains with a low of 9.17 ± 0.15 and a maximum of 11.74 ±0.15. Parental strains and F1s are indicated by black bars. Mice with the *B* and *D* haplotypes are indicated by blue and green bars, respectively. **(B)** *Ppp1r13b* maps as a highly significant *cis*-eQTL on Chr 12. The purple triangle indicates the location of *Ppp1r13b* within the mouse genome. **(C)** UCSC Genome Browser illustration of gene structure, and location of SNPs and InDels in *Ppp1r13b*. Reference Sequence mRNA is represented in blue. **(D)** Mice with the *D* haplotype of *Ppp1r13b* have significantly lower expression levels of the gene than those with the *B* haplotype (p = 1e-15). **(E)** In contrast, strains that carry the *D* haplotype of *Ppp1r13b* have a significantly greater number of necrotic axons than those with the *B* haplotype (p = 2.1e-3). **(F)** Terminal statistically significant categories taken from GO tree analyses of the correlates of *Ppp1r13b* in the retina. False discovery rates are shown next to each bar. The X-axis denotes the ratio of observed genes by the number of expected genes in the GO network.

**Figure 10:**
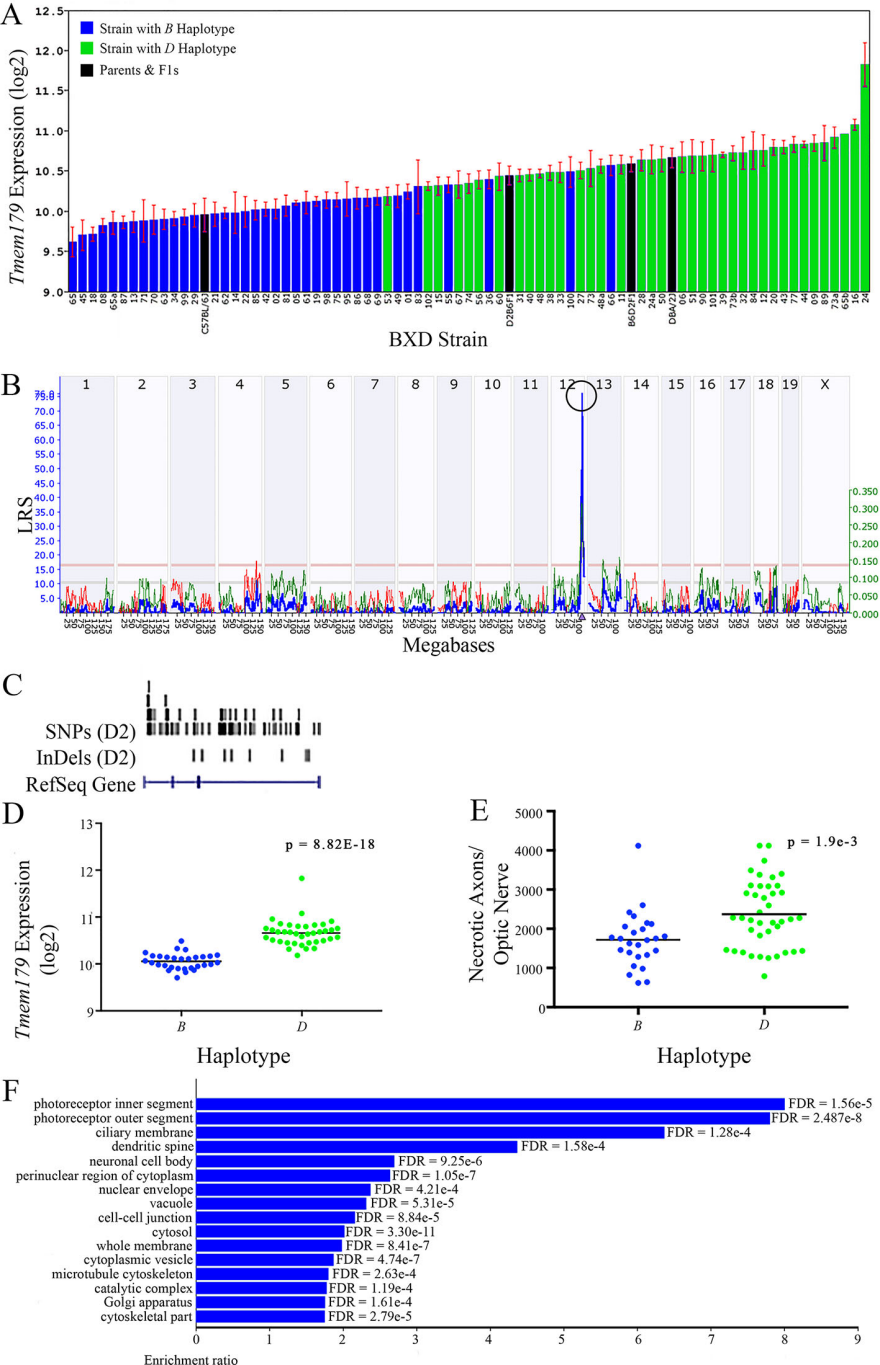
*Tmem179* as a candidate gene for modulation of optic nerve damage. **(A)** *Tmem179* expression in the retina varies across BXD strains. The bars depict a range of expression values from 9.62 ± 0.18 and 11.83 ± 0.27. On the Y-axis, *Tmem179* expression is on a Log2 scale. Parental strains and F1s are indicated by black bars. Mice with the *B* and *D* haplotypes are indicated by blue and green bars, respectively. **(B)** Genetic mapping revealed a single highly significant *cis*-eQTL for *Tmem179* on Chr 12. The purple triangle indicates the location of *Tmem179* within the mouse genome. **(C)** UCSC Genome Browser illustration of gene structure, and location of SNPs and InDels in *Tmem179*. Reference Sequence mRNA is represented in blue. **(D)** Mice with the *D* haplotype of *Tmem179* have significantly greater expression levels of the gene than those with the *B* haplotype (p = 8.82e-18). **(E)** Similarly, BXD strains that carry the *D* haplotype of *Tmem179* have a significantly greater number of necrotic axons than those with the B haplotype (p = 1.9e-3). **(F)** Terminal statistically significant categories taken from GO tree analyses of the correlates of *Tmem179* in the retina. False discovery rates are shown next to each bar. The X-axis denotes the ratio of observed genes by the number of expected genes in the GO network.

All of the eight positional gene candidates that remain after application of our selection criteria share several characteristics. For example, all candidates vary in expression by 2-fold to 6-fold (**Figures 3A**, **4A**, **5A**, **6A**, **7A**, **8A**, **9A**, and **10A**). Moreover, all candidates are cis-regulated (**Figures 3B**, **4B**, **5B**, **6B**, **7B**, **8B**, **9B** **, and** **10B**) and all contain multiple SNPs and InDels (**Figures 3C**, **4C**, **5C**, **6C**, **7C**, **8C**, **9C**, and **10C**). However, there are several differences among the candidates. Specifically, an increase in the expression levels of four genes is correlated with the *B* haplotype⟶ *Eif5*, *Bag5*, *Klc1,* and *Ppp1r13b* (**Figures 4D**, **5D**, **7D**, and **9D**, respectively)⟶while an increase in the expression levels of three genes is correlated with the *D* haplotype⟶i.e., *Apopt1, Xrcc3,* and *Tmem179* (**Figures 6D**, **8D**, and **10D**, respectively). The expression level of a ninth gene, *Cdc42bpb*, does not vary with the haplotype (**Figure 3D**). Interestingly, despite differences in expression levels that are associated with the *B* or *D* haplotypes, ON necrosis is significantly correlated with the *D* haplotype for all eight of the candidate genes (**Figures 3E**, **4E**, **5E**, **6E**, **7E**, **8E**, **9E**, and **10E**). These data suggest that in most instances, the *B* haplotype of the candidates is protective or that the *D* haplotype is harmful.

The cellular component functional networks within which the eight gene candidates function vary extensively. However, several shared categories are noted among the functional networks. Six of the candidates⟶*Cdc42bpb*, *Eif5, Bag5*, *Apopt1*, *Xrcc3,* and *Ppp1r13b*—function in a cellular component network that is associated with mitochondria (**Figures 3F**, **4F**, **5F**, **6F**, **8F**, and **9F**, respectively). Other shared networks include axons or cytoskeleton—*Cdc42bpb*, *Apopt1, Klc1*, *Xrcc3, Ppp1r13b,* and *Tmem179*, (**Figures 3F**, **6F**, **7F**, **8F**, **9F**, and **10F**, respectively). Association with both of these cellular structures strengthen these genes as plausible candidates. However, the pathways associated with two genes—*Xrcc3* and *Tmem179*—have their greatest number of genes associated with photoreceptor inner and outer segments (**Figures 8F** and **10F**), which diminishes their plausible role in ON health.

### Comparison of IOP and Necrotic Axons in the ON

**Figure 11:**
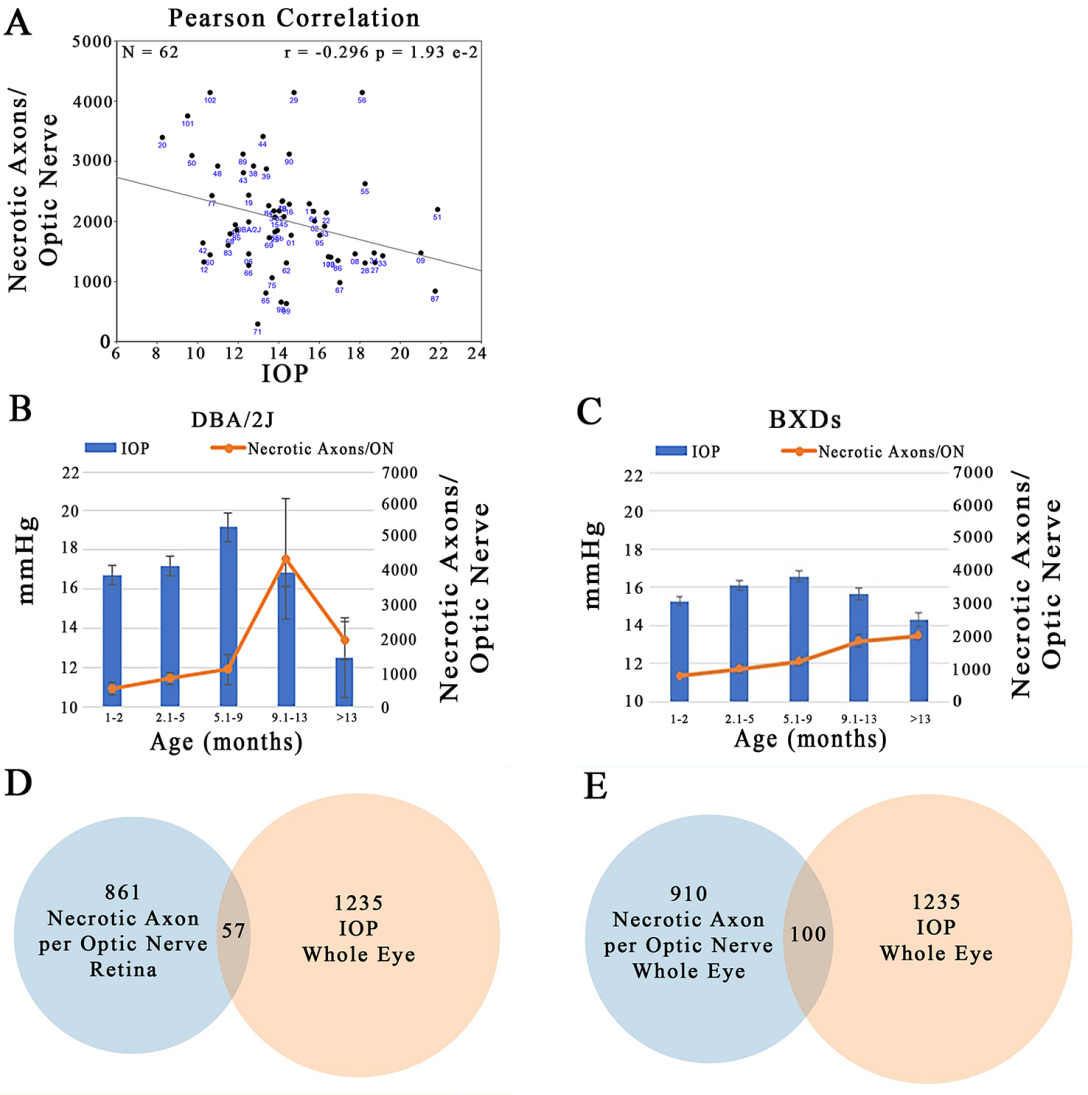
Independent regulation of IOP and ON necrosis. **(A)** IOP and the number of necrotic axons per ON are inversely correlated across the BXD family (r = -0.296; p=1.93e-2). **(B)** In D2 mice, the timing of peak IOP and ON necrosis do not correlate with IOP preceeding ON damage by ~4 months. **(C)** Across the BXD family, IOP peaks between 5-9 months of age. However, ON necrosis continues to increase throughout the life of mice. **(D)** From the list of the top 2000 correlates of IOP in the whole eye database, 1235 had expression levels > 6.8. Of the top 2000 correlates of ON necrosis in the retina database, 861 candidates were expressed above 6.8. After comparing the genes in both lists, only 57 were shared. **(E)** 1235 of the top 2000 correlates of IOP in the whole eye database were expressed > 6.8. 910 of the top 2000 correlates of ON necrosis in the whole eye database, were expressed above 6.8. Only 100 genes were shared among both lists.

Plotting IOP versus the number of necrotic axons in the ON across the BXD family at >13 months of age illustrates that there is a large degree of scatter in the data. Despite this, there is a significant negative correlation between the two traits (**Figure 11A**; r = –0.296; p = 1.93 e-2). Specifically an elevation in IOP is associated with fewer necrotic axons in the ON. This is contrary to the expected outcome, suggesting that in this genetic reference panel, the two traits most associated with glaucoma are regulated independently of each other. A plausible confounding factor in the association of these two traits may lie in the difference timing of the peak IOP and ON necrosis for each strain. For example, we demonstrate that in the D2 parent, IOP peaks between 5.1 and 9 months, yet the number of necrotic axons peaks several months later at 9–13 months (**Figure 11B**), similar to that demonstrated by other groups (Anderson et al., 2002; Libby et al., 2005b; Williams et al., 2017). In contrast, across the BXD family, although IOP peaks at the same age group as in D2, ON necrosis continues to increase throughout the life of the mouse (**Figure 11C**). It is also important to note that on average, the BXD family has lower IOP and ON necrosis than the D2 parent. In further support of this discord between IOP and ON necrosis, less than 10% of the genes are shared between IOP and the number of necrotic axons in the ON in mice aged more than 13 months (**Figures 11D, E**; listed in **Supplemental Tables 3** and **4**).

## Discussion

Damage to the ON, which is comprised of the axons of RGCs, leads to loss of visual field in glaucoma. Across multiple populations, including humans and mice, there are several risk factors that are associated with and predictive for ON damage. The greatest risk factors have been reported to be elevated IOP and reduced CCT (Gordon et al., 2002). Moreover, in the D2 parent mutations in *Tyrp1* and *Gpnmb* have been reported as being causative for the pigmentary dispersion glaucoma that leads to ON damage (John et al., 1998). The locus that we identified as a modulator of the number of necrotic axons in the ON maps is distinct from the loci that modulate IOP, CCT, *Tyrp1* and *Gpnmb,* suggesting that our newly identified locus appears to be regulated independently of the previously identified gene modulators of glaucoma-associated endophenotypes.

Using a modification of the criteria we used to identify a novel modulator of IOP (Chintalapudi et al., 2017), we narrowed the list of 156 positional candidates in our Chr 12 locus to eight viable candidates: *Cdc42bpb; Eif5; Bag5; Apopt1; Klc1; Xrcc3*; *Ppp1r13b,* and *Tmem179*. All eight genes are cis-modulated, correlate with the variation in the number of necrotic axons in the optic nerve, are expressed in the retina and/or eye, and harbor sequence variants. Seven of the candidate genes function in cellular component networks associated with mitochondria. Because preservation of mitochondrial health has been demonstrated to prevent and even reverse ON damage (Williams et al., 2017), these genes remain as high priority candidates. Two of the eight candidates function in networks that contains multiple genes associated with photoreceptors. This finding lessens the likelihood that they are strong candidates for modulation of ON necrosis. A caveat would be if localization studies indicate that the gene products are also found in the vicinity of retinal ganglion cell axons or the ON. However, this is yet to be determined. Lastly, one positional candidate—*Wdr25*—fulfilled six of seven inclusion criteria. While this one gene cannot be excluded completely, it is a lower priority candidates than the other eight genes that fulfill all of our inclusion criteria. It will be pursued in a second tier analysis. No additional positional candidates were identified after the requirement for cis-modulation was removed. While it is theoretically possible that a non-cis-modulated gene could have a driving effect in the development of optic nerve degeneration, it is much more likely that it is part of the downstream changes of the true gene modulator of the endophenotype. With several positional candidates fulfilling all of our inclusion criteria, it is vastly more likely that one of them is the driver for the development of glaucoma that we documented in the BXD family.

It is important to note that some of our positional candidates—*Eif5, Bag5, Klc1, and Ppp1r13b*—have been previously demonstrated to play a role in glaucoma and/or RGC health (Elluru et al., 1995; Butowt and Von Bartheld, 2007; Guo et al., 2011; Rogers et al., 2012; Wilson et al., 2013; Wang et al., 2014). Although these findings further strengthen several of our candidates, additional investigations are required to further narrow the list of eight candidate genes that modulate the progression from healthy and fully functional retinal ganglion cell axons to necrosis. To rule out additional candidates, localized expression would need to be visualized with immunohistochemistry and/or *in situ* hybridization. To remain as a viable candidate, the gene product should be expressed in retinal ganglion cells, their axons within the ON or in the surrounding space and/or invading neuroinflammatory cells. In contrast, if the protein is localized exclusively to a structure that is distant from the ON, it is far less likely to play a role in its health. Additional studies that would facilitate removal of a gene candidate from the short list of plausible modulators of axon necrosis include gene knockout studies, CRISPR modifications, and/or transgression of the polymorphic gene onto a different genetic background followed by an evaluation of the resulting phenotypes.

Because IOP is the primary risk factor for visual field loss in glaucoma, we sought to determine the association of ON damage and IOP across the BXD family at greater than 13 months of age. Our findings illustrated that there is minimal correlation between the two traits, and contrary to expectations, the relationship is inverse with strains having the highest IOP values having fewer necrotic axons in the ON. A caveat may lie in the differential timing of these two traits. As we illustrate in the D2 parent, the peak IOP precedes the peak of ON necrosis by approximately four months. In contrast, although both IOP and ON necrosis increase with age across the BXD genetic reference panel, ON damage continues to increase in each subsequent age group. These data strongly suggest that the two most studied endophenotypes of glaucoma—IOP and ON damage—are autonomously modulated and/or a sliding time scale may need to be incorporated into their combined analyses. Furthermore, the recombination of the B6 and D2 genomes has further complicated the statistical analysis of these glaucoma phenotypes in the complex BXD family.

In summary, we used systems genetics and the BXD GRP to identify a narrow genomic region on Chr 12 that modulates the number of necrotic axons in the ON in aged mice. This locus was not present in data obtained from young mice, suggesting that the locus also has an aging component. Using our stringent in silico criteria, we have narrowed the list of positional candidates from 156 to eight. Additional studies are required to further narrow the list to the causative gene modulator of ON necrosis. We also determined that in elderly mice, IOP and ON necrosis appear to be independently controlled. Future analyses should take into account a differential time scale of IOP and ON damage. Our study suggests that as treatment strategies for glaucoma are developed, multiple therapeutic targets should be identified and evaluated.

## Data Availability Statement

The datasets generated for this study are available on request to the corresponding author.

## Ethics Statement

All procedures involving mice were approved by the Animal Care and Use review board of UTHSC and followed the Association of Research in Vision and Ophthalmology (ARVO) Statement for the Use of Animals in Ophthalmic and Vision Research, in addition to the guidelines for laboratory animal experiments (Institute of Laboratory Animal Resources, Public Health Service Policy on Humane Care and Use of Laboratory Animals).

## Author Contributions

LL, RW and MJ conceived the experiments. AS, ES, RS, LL, and MJ conducted experiments. AS, ES, RS, LL, RW and MJ participated in data interpretation and discussion. LL, RW, and MJ conceptualized the project, provided resources and supervised the experiments for completion of the study. AS, RW and MJ wrote the manuscript. All authors reviewed and contributed intellectually to the article.

## Funding

This study received financial support from: the Center for Integrative and Translational Genomics at the University of Tennessee Health Science Center; NEI Grants R01EY021200, and P30EY013080; NIAAA Grant U01AA01666; and an unrestricted grant from Research to Prevent Blindness, Inc.

## Conflict of Interest

The authors declare that the research was conducted in the absence of any commercial or financial relationships that could be construed as a potential conflict of interest.
