# Supplementary figures and images for: Systems Genetics of Optic Nerve Axon Necrosis During Glaucoma
Source: Front Genet. 2020 Feb 27;11:31. doi: 10.3389/fgene.2020.00031 (PMC7056908; doi:10.3389/fgene.2020.00031)

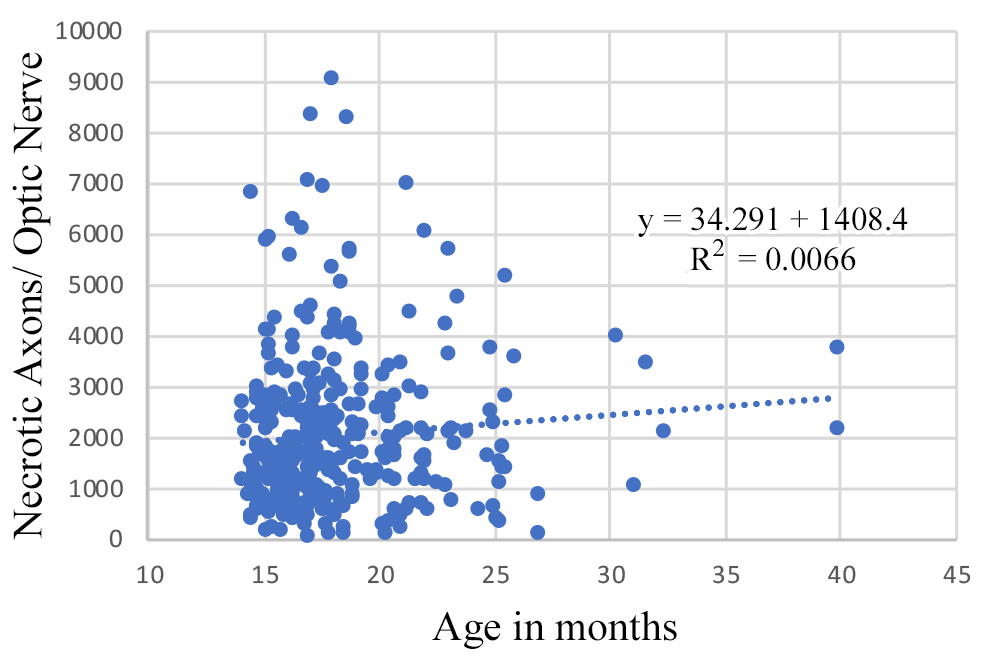

Supplement: Supplementary file 1 [file Image_1.tif]

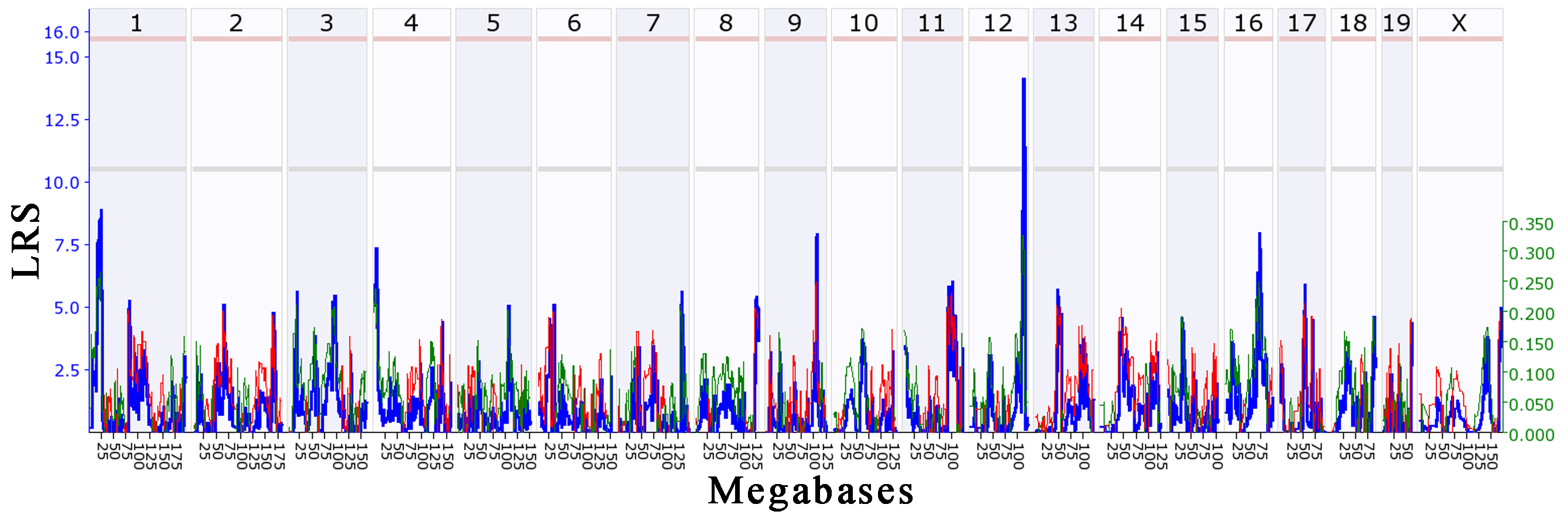

Supplement: Supplementary file 2 [file Image_2.tif]
